# Carotid sinus syndrome as the presenting symptom of cystadenolymphoma

**DOI:** 10.1186/1746-160X-8-31

**Published:** 2012-11-14

**Authors:** Nelson Noroozi, Ali Modabber, Frank Hölzle, Till Braunschweig, Dieter Riediger, Marcus Gerressen, Alireza Ghassemi

**Affiliations:** 1 Department of Oral, Maxillofacial and Plastic Facial Surgery, University Hospital RWTH-Aachen, Pauwelsstraße 30, Aachen, 52074, Germany; 2 Institute of Pathology, University Hospital RWTH-Aachen, Aachen, Germany; 3 Department of Oral, Maxillofacial and Plastic Facial Surgery, Heinrich-Braun Hospital Zwickau, Zwickau, Germany

**Keywords:** Cystadenolymphoma, Warthin’s tumor, Parotid gland tumors, Syncope, Carotid sinus syndrome

## Abstract

Carotid sinus syndrome is a serious manifestation of head and neck malignancy. The purpose of this study was to clarify the presence of carotid sinus syndrome in a patient with cystadenolymphoma. To our knowledge carotid sinus syndrome secondary to cystadenolymphoma has not been reported to date. A 45-year-old woman with one-week-old swelling in the left mandibular angle having disturbing symptoms of vertigo, consciousness and sinus arrest. Holter monitoring revealed several episodes of sinus arrest. Ultrasonography showed a well-defined space-occupying lesion of about 31 mm in length and 17 mm in width located in the deep lobe of the left parotid gland. Computerized tomography (CT) showed a large mass extending into the carotid space and protruding into the parapharyngeal space. Parotidectomy was performed. Surgical removal of the tumor resulted in complete amelioration of symptoms and disappearance of electrocardiogram abnormalities. Here we report on a clinical case of carotid sinus syndrome associated with cystadenolymphoma. To our knowledge carotid sinus syndrome secondary to cystadenolymphoma has not been reported to date, and is made more remarkable as a possible differential diagnosis after clarification of all possible causes. Early diagnosis and immediate management can minimize complications.

## Background

Carotid sinus syndrome is a rare but serious manifestation of head and neck malignancy [1]. Although a number of different types of the syndrome have been proposed by Weiss and Baker [2], it is not always easy to fit a patient into these categories. However, to our knowledge, this is the first report of cystadenolymphoma presenting as recurrent syncopal episodes, and the surgical removal of the tumor resulted in complete amelioration of symptoms and disappearance of electrocardiogram abnormalities.

## Case report

A 45-year-old woman with one-week-old swelling in the left mandibular angle was admitted to our oral and maxillofacial surgery department with the complaints of recurrent episodes of vertigo and consciousness. The vertigos gradually increased in frequency and intensity since they started one month prior her admission. Physical examination revealed a discrete raised, painless mass occupying the left parotid area witch adhered to underlying soft tissue, extending approximately 2 cm in diameter in the sub- and retromandibular regions. There were no restrictions on the cranial nerves supplying this area. No odontogenic cause or any other inflammatory factors could be responsible for the parotid gland swelling. There was no palpable lymphadenopathy in the left neck. Blood pressure was 115/62 mm Hg and the heart rate was 63 beats per minute. Routine laboratory investigations were normal. 24-hour Holter monitoring revealed several episodes of sinus arrest. Ultrasonography showed a well-defined space-occupying lesion of about 31 mm in length and 17 mm in width, which was hypo echoic and located in the deep lobe of the left parotid gland. The rest of the gland showed a homogeneous parenchyma.

**Figure 1:**
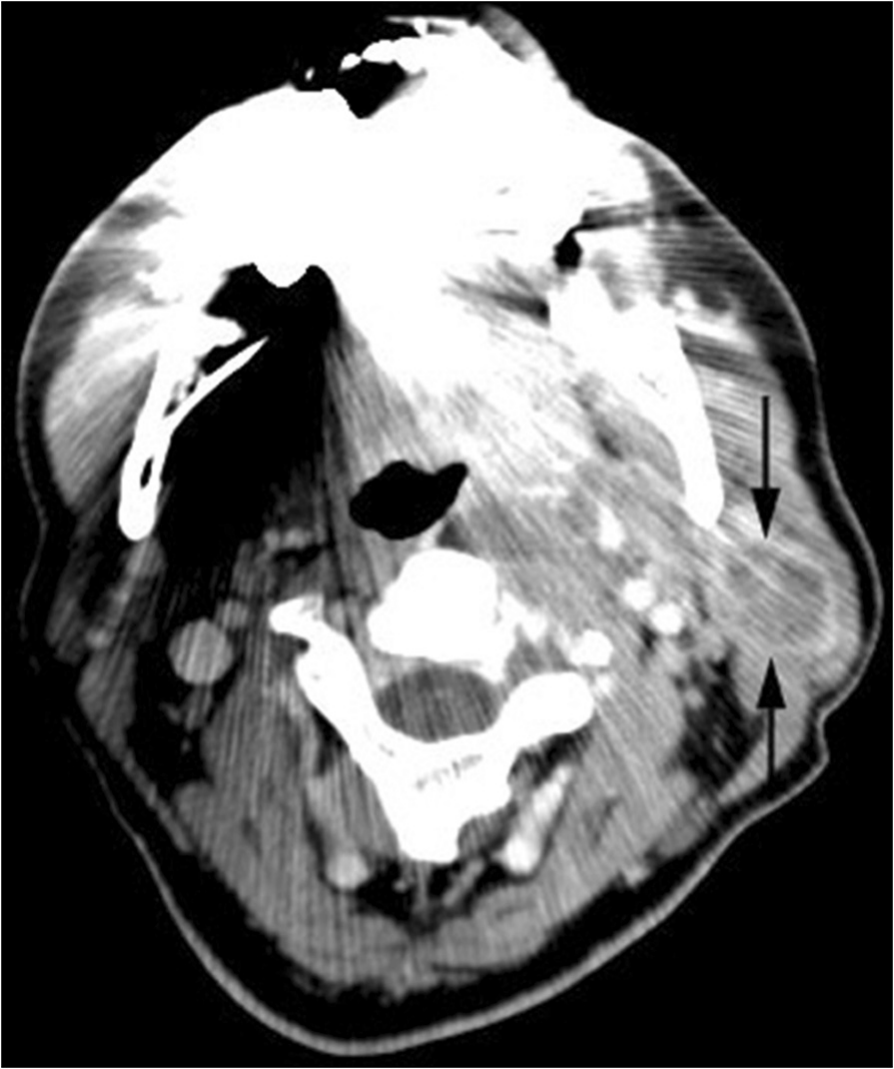
Computerized tomography (CT) at the level of the submandibular area demonstrating the mass of the left parotid gland extending into the carotid space.

Computerized tomography (CT) showed a large mass extending into the carotid space and protruding into the parapharyngeal space (Figure 1).

**Figure 2:**
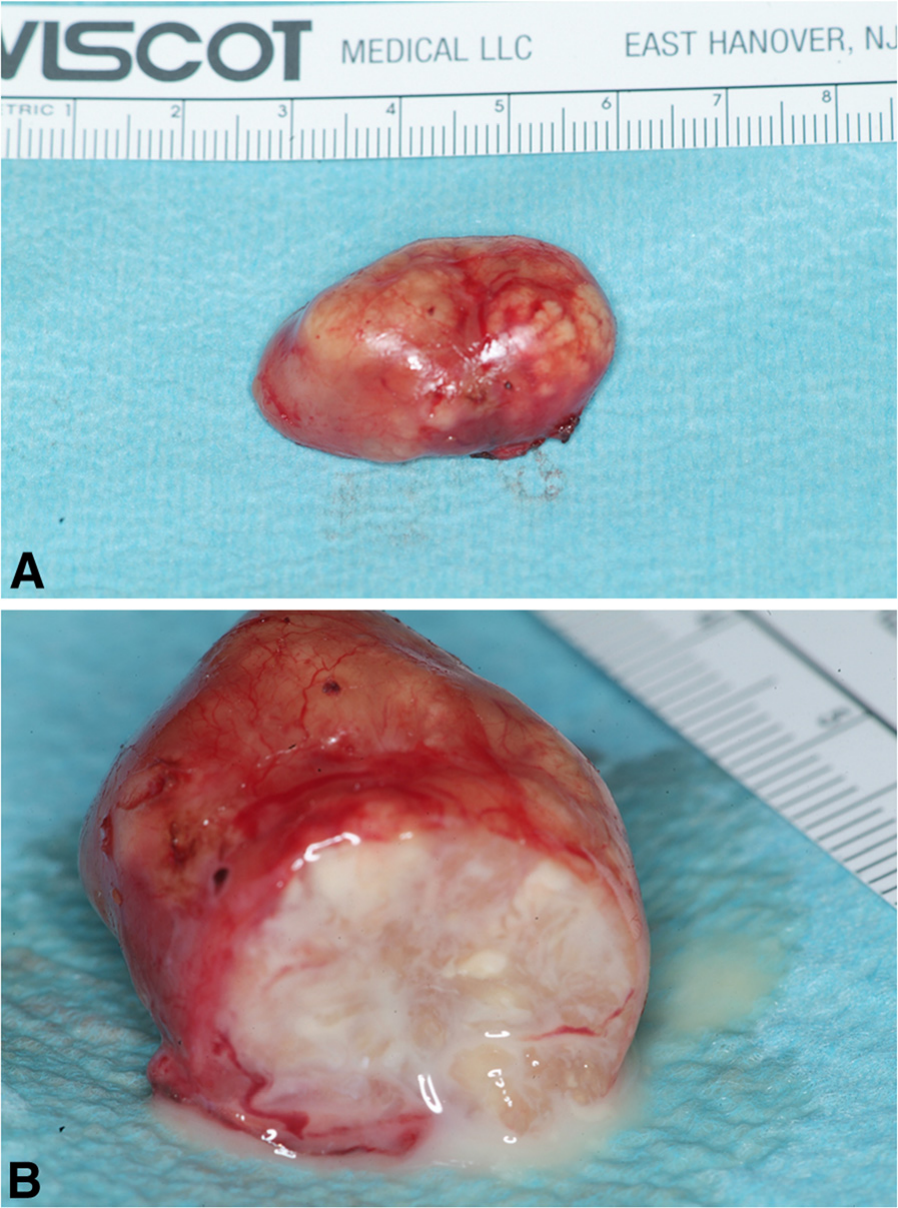
**A/B: 2A:The tumor was oval-shaped, encapsulated, and partially nodular without any regional lymph gland involvement.2B**: Upon opening the mass viscous, white-colored fluid was released.

A left parotidectomy was performed. The resected gland was a hard mass, which had spread over the bifurcation of the left carotid artery without invading the vessel wall itself. The tumor was oval-shaped, encapsulated, and partially nodular without any regional lymph gland involvement. Upon opening the mass viscous, white-colored fluid was released (Figure 2A/B).

**Figure 3:**
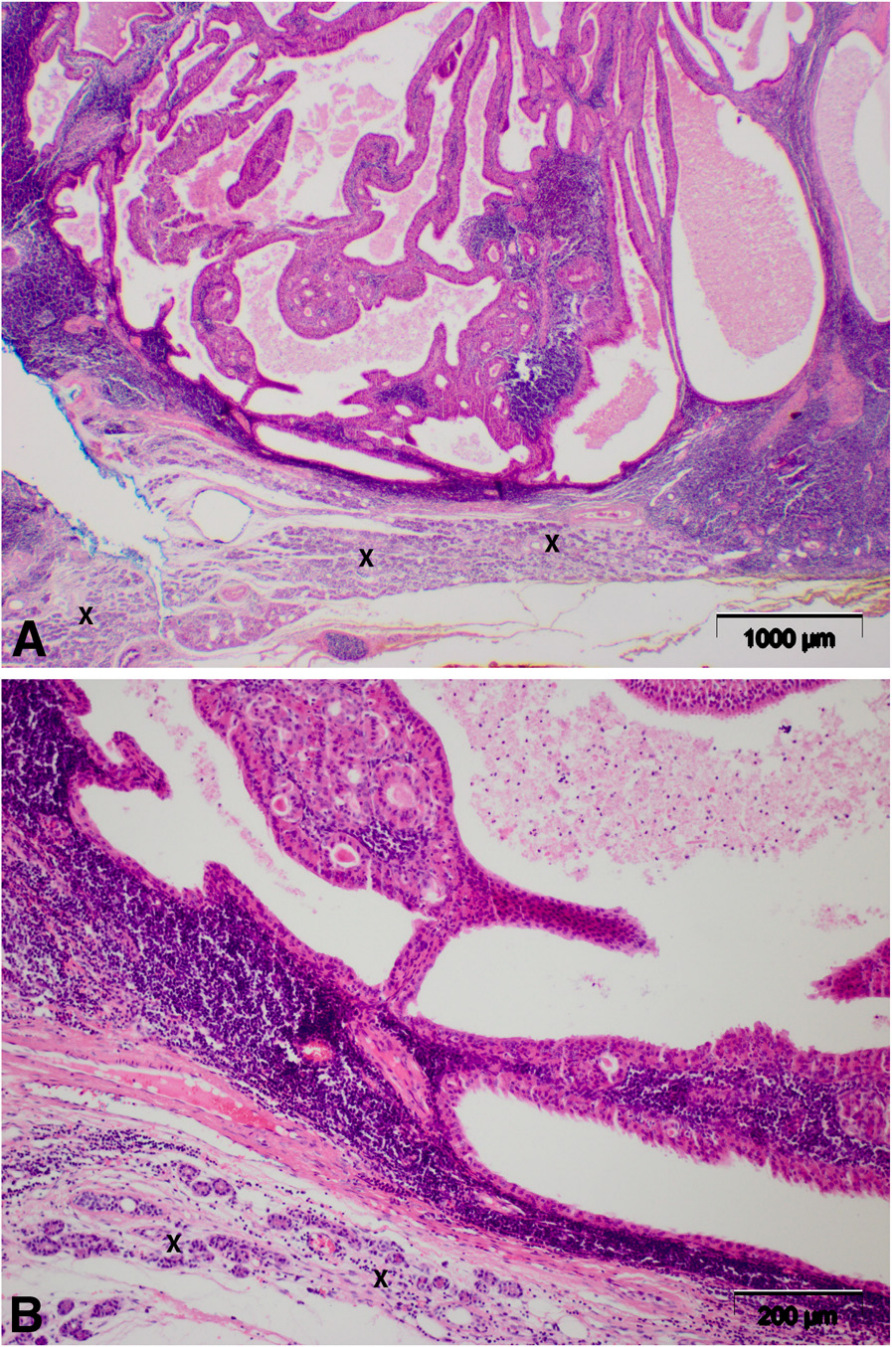
**A/B: Hematoxylin-eosin (H&E) parotid gland (x), adjacent lymph nodes with glandular-epithelial formations.** (**3A**: original magnification x25; **3B**: original magnification x100).

The pathology report showed a lymphoid tissue with multifocal double-row hypereosinophilic epithelium with retention of thickened secretions in the lumen as well as squamous cell metaplasia consistent with typical parotid gland cystadenolymphoma (Figure 3A/B).

The patient started ambulating from the second postoperative day without any symptoms of vertigo and consciousness. 24-hour Holter monitoring was repeated on three occasions at weekly intervals and revealed regular sinus rhythm at a rate of 80 to 90 per minute. 6 months postoperatively, the patient was asymptomatic.

## Discussion and conclusion

The carotid sinus, located just superior to the bifurcation of the carotid artery controls the heart rate and blood pressure via receptors located within the arterial adventitia. The afferent limb of the carotid sinus reflex begins with these receptors [3]. Myelinated nerve fibers emerge from these menisci as spiral fibers and from the sinus nerve of Hering, a branch of the glossopharyngeal nerve. Other fibers may follow the hypoglossal nerve, vagus nerve or cervical sympathetic nerves to the medulla. The efferent fibers descend in the vagus and cervical sympathetic nerves to the cardioinhibitory and vasomotor centers [4,5]. The carotid sinus syndrome consists of a cardiovascular symptom complex resulting from excitation of a hyperactive carotid sinus reflex. Weiss and Baker [2] classified three types of responses leading to carotid sinus stimulation. Cardioinhibitory response, which is expressed as bradycardia and asystole, vasodepressor response, characterized by profound hypotension without bradycardia, and cerebral response, which is an interference with the circulation of the ipsilateral cerebral hemisphere circulation [6]. The pathophysiology of carotid sinus syndrome secondary to head and neck malignancy is not well understood. Local pathologic conditions adjacent to the carotid sinus such as enlarged lymph nodes, operation scars, and mechanical pressure by a mass on carotid sinus or by actual invasion of the carotid sinus, sinus nerve or glossopharyngeal nerve by tumor have been postulated to produce carotid sinus syndrome [1,4,7,8].

The tumor may cause either a spontaneous abnormal afferent discharge in the damaged nerve itself or may lead to ephaptic conduction, either between glossopharyngeal efferent motor fibers and afferent sinus sensory fibers or between the glossopharyngeal nerve and other nearby nerves, inducing an abnormally strong carotid sinus reflex. It also been postulated that compression of the nerve outside of the carotid sinus reflex arc, such as the glossopharyngeal nerve, can cause carotid sinus syndrome [9-13].

It is more likely that head and neck tumors cause syncope by involvement of the glossopharyngeal or vagus nerve in those patients in whom carotid sinus massage does not induce syncope.

The majority of reports of carotid sinus syndrome associated with head and neck malignancy relate to extensive nodal involvement in the neck [14].

The immediate treatment of carotid sinus syndrome includes anticholinergic medications and cardiac pacing. Surgery is reserved for those who fail medical therapy [15]. Radiation therapy is occasionally beneficial [16].

In our presented case the disturbing symptoms of vertigo, consciousness and sinus arrest, all disappeared following resection of the parotid tumor. We postulate that the tumor produced sustained compression on the carotid sinus resulting in carotid sinus syndrome. The patient was followed postoperatively for 6 months without any clinical symptoms. In summary, this is the first report of carotid sinus syndrome exacerbated by a benign parotid tumor and is made more remarkable here as a possible differential diagnosis after clarification of all possible causes. Early diagnosis and immediate management can minimize complications. Resection of the tumor resulted in amelioration of symptoms and disappearance of electrocardiography abnormalities.

## Consent statement

Written informed consent was obtained from the patient for publication of this case report and accompanying images. A copy of the written consent is available for review by the Editor-in-Chief of this journal.

## Competing interests

The authors declare that they have no competing interests.

## Authors' contributions

NN, AM, FH, TB, DR, MG and AG conceived of the study and participated in its design and coordination. NN and AM drafted the manuscript and contributed equally to this work. NN, AM, MG and AG were involved in revising the manuscript. All authors read and approved the final manuscript.
